# Epigenetic modifications of the VGF gene in human non-small cell lung cancer tissues pave the way towards enhanced expression

**DOI:** 10.1186/s13148-017-0423-6

**Published:** 2017-11-28

**Authors:** Sebastian Marwitz, Lena Heinbockel, Swetlana Scheufele, Dörte Nitschkowski, Christian Kugler, Sven Perner, Martin Reck, Ole Ammerpohl, Torsten Goldmann

**Affiliations:** 1 Pathology of the University Medical Center Schleswig-Holstein (UKSH), Campus Lübeck and the Research Center Borstel Parkallee 3, 23845 Borstel, Germany; 2 Institute of Human Genetics, University Medical Center Schleswig-Holstein (UKSH), D-24105 Kiel, Germany; 3 Surgery, LungenClinic Grosshansdorf, D-22927 Grosshansdorf, Germany; 4 Oncology, LungenClinic Grosshansdorf, D-22927 Grosshansdorf, Germany; 5 Institute of Human Genetics, University Medical Center Ulm, D-89081 Ulm, Germany; 6 Airway Research Center North, German Center for Lung Research (DZL), D-22927 Großhansdorf, Germany

**Keywords:** VGF, Methylome, Transcriptome, Non-small cell lung cancer, NSCLC

## Abstract

Hwang et al. recently showed that VGF substantially contributes to the resistance of human lung cancer cells towards epidermal growth factor receptor kinase inhibitors. This was further linked to enhanced epithelial–mesenchymal transition. Here, we demonstrate that VGF is epigenetically modified in non-small cell lung cancer tissues compared to corresponding tumor-free lung tissues from the same donors by using methylome bead chip analyses. These epigenetic modifications trigger an increased transcription of the VGF gene within the tumors, which then leads to an increased expression of the protein, facilitating epithelial–mesenchymal transition, and the resistance to kinase inhibitors. These results should be taken into account in the design of novel therapeutic and diagnostic approaches.

Hwang et al. recently demonstrated impressively that VGF is playing a crucial role in the resistance of lung cancer cells to epidermal growth factor receptor (EGFR) kinase inhibitors [1]. Moreover, it was shown that there was a correlation of VGF expression with poor survival and the authors clearly showed a connection of VGF and epithelial–mesenchymal transition (EMT). Up to date, there is no data on the methylation status of the VGF gene in lung cancer, which could be a possible epigenetic base of the observed enhanced expression.

In continuation of recently published work focusing on the role of the TGFß pseudo-receptor BAMBI with regard to EMT in non-small-cell lung cancer (NSCLC), we analyzed VGF in tumors and corresponding tumor-free tissues in a cohort of NSCLC patients [2]. Utilization of patient material was approved by the ethics council at the University of Lübeck (AZ-12-220).

Here, we investigated potential epigenetic modifications of the *VGF* gene in 33 patients (20 males and 13 females) matched controls by Infinium HumanMethylation450k BeadChips (Illumina Inc., San Diego, CA, USA) as previously described [3]. All tumors were primary NSCLC. The cohort comprised 15 adenocarcinomas and 18 squamous cell carcinomas of the lung; the mean age of the patients at surgery was 65.6 years. Eighteen out of these 33 patients were analyzed by transcriptome analysis as recently shown [2]. Quantile-normalized relative gene expression values for VGF were extracted from the GEO-dataset GSE74706 and analyzed with GraphPad Prism v.7. Furthermore, the expression of VGF was analyzed on the protein level by immunohistochemistry utilizing a polyclonal antibody (ab 115609, Abcam, Cambridge, UK) on tissue microarrays of formalin-fixed, paraffin-embedded tissues from 25 NSCLC cases (13 adenocarcinomas/12 squamous cell carcinomas) and matched controls as described elsewhere [2].

Hierarchical clustering of the methylation level of several VGF CpG loci as obtained by GenomeStudio software and visualized by OMICS Explorer 2.1 (Fig. 1a) exhibits clear differentiation between the tumor-free tissues and the tumors. Of the 16 VGF-related CpG loci present on the HumanMethylation450 BeadChip, six are located in the gene body, five in the TSS1500 (a region beginning at the transcription start site and ranging 1500 bp upstream), two in the TSS200, two in the 5′UTR, and one each in the 3′UTR and the first exon (cg21186299 is annotated to 1st exon as well the 5′UTR). Nine CpG loci are located in regions with known or predicted enhancer activity while six loci are located in DNaseI hypersensitive sites according to the annotation provided by Illumina. Strongest differences in the mean beta values (> 10%) have been found in cg08097755, cg05225187, and cg12135573 located in the 5′UTR and the southern shore of a CpG island located in the VGF gene.

**Fig. 1:**
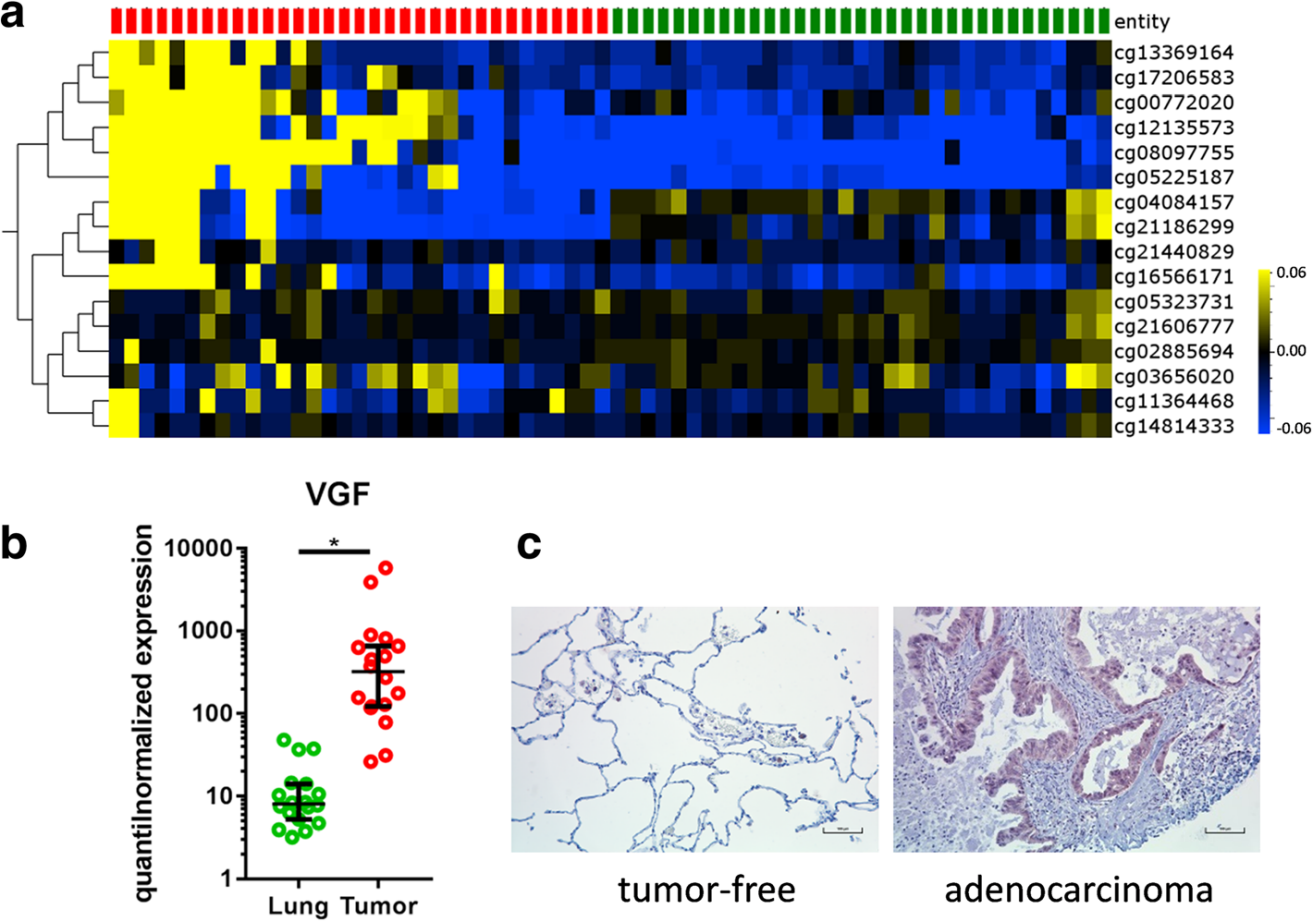
VGF is differentially methylated and expressed in tumor-free lung tissues and NSCLC tissues. Normalized methylation analysis of CpG loci for *VGF* present on the HumanMethylation450K BeadChip with tumor samples indicated in red and matched tumor-free controls indicated in green (heatmap: *yellow* high, *blue* low DNA methylation values; mean DNA methylation = 0) (**a**). Relative gene expression level of *VGF* as quantile-normalized expression values of tumor-free lungs and matched tumors depicting the median with the 95% confidence interval as error bars. Paired two-sided *T* test with *p* ≤ 0.05 (*) regarded significant was used for statistical analysis (**b**). Exemplary results from IHC analysis showing VGF expression on protein level in a selected patient tissue (**c**)

Furthermore, a clearly increased transcription of VGF in the tumors is observed on the RNA level as compared to corresponding tumor-free tissues (Fig. 1b). These results were confirmed on the protein level, where an elevated expression of VGF was observed in the tumors (Fig. 1c).

These findings suggest a biological implication of these epigenetic modifications taking place in NSCLC development and, hence, paving the way towards an enhanced expression, which then results in enhanced EMT facilitating resistance against EGFR kinase inhibitors as described by Hwang et al. [1].

The epigenetic imprinting taking place in NSCLC regarding the modifications of VGF could be taken into account in therapeutic and diagnostic approaches. Taken together, we show that VGF is epigenetically modified in human NSCLC tissues if compared to tumor-free lung tissues, which results in an increased transcription and protein expression.

## References

[1] Hwang W, Chiu YF, Kuo MH, Lee KL, Lee AC, CC Y (2017). Expression of neuroendocrine factor VGF in lung cancer cells confers resistance to EGFR kinase inhibitors and triggers epithelial-to-mesenchymal transition. Cancer Res.

[2] Marwitz S, Depner S, Dvornikov D, Merkle R, Szczygieł M, Müller-Decker K (2016). Downregulation of the TGFβ pseudoreceptor BAMBI in non-small cell lung cancer enhances TGFβ signaling and invasion. Cancer Res.

[3] Marwitz S, Kolarova J, Reck M, Reinmuth N, Kugler C, Schädlich I (2014). The tissue is the issue: improved methylome analysis from paraffin-embedded tissues by application of the HOPE technique. Lab Investig.

